# Epstein-Barr virus BARF1-induced NFκB/miR-146a/SMAD4 alterations in stomach cancer cells

**DOI:** 10.18632/oncotarget.10511

**Published:** 2016-07-09

**Authors:** Dong Ha Kim, Mee Soo Chang, Chan Jin Yoon, Jaap M. Middeldorp, Olivia M. Martinez, Sun-ju Byeon, Sun Young Rha, Sung Han Kim, Yang Soo Kim, Jun Hee Woo

**Affiliations:** ^1^ Asan Institute for Life Sciences, Department of Infectious Diseases, Asan Medical Center, University of Ulsan College of Medicine, Seoul, Republic of Korea; ^2^ Department of Infectious Diseases, Asan Medical Center, University of Ulsan College of Medicine, Seoul, Republic of Korea; ^3^ Department of Pathology, Seoul National University Boramae Hospital, Seoul National University College of Medicine, Seoul, Republic of Korea; ^4^ Department of Pathology, VU University Medical Center, Amsterdam, the Netherlands; ^5^ Department of Surgery/Division of Abdominal Transplantation, Stanford University School of Medicine, Stanford, CA, USA; ^6^ Department of Internal Medicine, Yonsei University College of Medicine, Seoul, Republic of Korea

**Keywords:** Epstein-Barr virus, BARF1, NFκB, miR-146a, SMAD4

## Abstract

Epstein-Barr virus (EBV)-encoded BamHI-A rightward frame 1 (BARF1) is a putative viral oncogene in EBV-infected stomach cancer. The aim of the present study was to investigate BARF1-induced cellular protein and microRNA alterations. In this study, BARF1-expressing stomach cancer cells showed a high rate of proliferation, high levels of NFκB, and miR-146a upregulation, which was reversed by NFκB knockdown. During BARF1-induced NFκB upregulation, hCSF1 receptor level was unchanged. Knockdown of BARF1 in the naturally EBV-infected YCCEL1 stomach cancer cells suppressed cell proliferation, and downregulated NFκB and miR-146a. SMAD4 was identified as a miR-146a target and was downregulated in BARF1-expressing cells, whereas SMAD4 expression was restored by anti-miR-146a. Knockdown of BARF1 in YCCEL1 cells upregulated SMAD4, and this effect was reversed by miR-146a overexpression. Transfection of BARF1-expressing cells with pCEP4-SMAD4 abolished the cell proliferating effect of BARF1. In stomach cancer tissues, miR-146a was expressed at higher levels, and more frequent NFκB nuclear positivity immunohistochemically, but not of SMAD4 nuclear loss was found in the EBV-positive group compared with the EBV-negative group. In conclusion, EBV-encoded BARF1 promotes cell proliferation in stomach cancer by upregulating NFκB and miR-146a and downregulating SMAD4, thereby contributing to EBV-induced stomach cancer progression.

## INTRODUCTION

In 2014, molecular analytic data from The Cancer Genome Atlas (TCGA) (http://cbioportal.org) corroborated that Epstein-Barr virus (EBV)-associated stomach cancer is a specialized subset of stomach cancer [1]. EBV is responsible for various human lymphoid and epithelial malignancies [1, 2, 3] including EBV-infected stomach cancer, which was first reported in 1990 [4]. Currently, stomach cancer is the most frequent EBV-associated malignancy [5, 6]. Stomach cancer caused by EBV infection accounts for approximately 5–10% of all stomach cancers worldwide irrespective of cancer incidence [1, 5–13]. Over the last 25 years, accumulating evidence has shown that EBV infection may directly contribute to the development of stomach cancer. EBV-positive gastric carcinomas are characterized by the monoclonal proliferation of EBV-infected cancer cells [14], global CpG island methylation of cancer-related genes [15], unique methylation patterns leading to CDKN2A (p16) downregulation [1, 16], and hyperactive T-cell activation [1, 11]. In addition, EBV-positive stomach cancer shows characteristic clinicopathological features, including a higher prevalence in male patients, predominant localization to the proximal stomach, a tendency towards a poorly differentiated histologic type and a diffuse Lauren-type, the presence of lymphoid stroma [1, 9–11], and a unique cellular protein expression profile [12].

The mechanism by which EBV causes stomach cancer remains unclear. EBV-encoded BamHI-A rightward frame 1 (BARF1) was suggested to function as a viral oncogene (oncogenic initiator or oncogenic cofactor) in EBV-positive stomach cancer [5, 17–19]. It has been demonstrated that BARF1 exists in all of EBV-positive stomach cancer tissues with a specialized BARF1-nucleic acid sequence-based amplification (NASBA) method using frozen tissue [19]. Wei et al first described recombinant BARF1-induced oncogenic activities, such as the tumorigenic transformation of mouse fibroblasts and tumor formation in new-born rats [20]. BARF1 has sequence homology with colony stimulating factor-1 receptor (hCSF1 receptor), and BARF1 binds to hCSF1 (macrophage-colony stimulating factor), similar to the binding between hCSF1 and hCSF1 receptor, which modulates the fates of immune-related cells such as macrophages [18, 21, 22]. EBV-encoded latent membrane protein (LMP) 2A is expressed on almost all EBV-positive human cancers [23], and the role of LMP2A in EBV-induced stomach carcinogenesis has been analyzed [6, 10, 24, 25]. LMP1 is an established viral oncogene in EBV-infected malignant lymphoma and nasopharyngeal cancer [3, 26]; however, LMP1 is not expressed in EBV-infected stomach cancer due to promoter methylation [3, 6, 10].

MicroRNAs (miRNAs) are endogenous small (19–22 nucleotides) non-coding RNAs that function as post-transcriptional regulators of gene expression by binding to complementary sites in the 3′ untranslated region (3′ UTR) of target mRNAs [27, 28]. miRNAs have been implicated in the regulation of various biological processes such as inflammation, infection, immune responses and tumorigenesis [28–30]. The established viral oncogene LMP1 upregulates several cellular miRNAs in different human malignancies [31–36]. To the best of our knowledge, BARF1-induced cellular miRNA changes have not yet to be observed in human malignancies. We previously showed that secreted BARF1 upregulated nuclear factor κB (NFκB) in an autocrine and paracrine manner in stomach cancer [5]. NFκB induces miR-146a expression, and the promoter region of miR-146a contains NFκB binding sites [29, 33, 37, 38]. Furthermore, miR-146a can directly downregulate several genes including ‘similar to mothers against decapentaplegic homologue 4′ (SMAD4) [39, 40], STAT-1, and IRF-5 [29]. Of these, the SMAD4 protein is related to NFκB activity [41, 42]. SMAD4 is a central mediator of the transforming growth factor beta (TGFβ) signaling pathway. In this pathway, TGFβ activation leads to the formation of a heteromeric complex between activated SMAD2/SMAD3 and SMAD4, which translocates into the nucleus, and affects transcriptional activity [43–45]. The involvement of SMAD proteins in EBV-associated oncogenesis has been described previously. EBV-encoded LMP1 antagonizes the TGFβ-SMAD pathway through NFκB signaling [41, 46], and EBV-encoded EBNA1 suppresses the interaction of SMAD2 with SMAD4 [47].

The objective of the present study was to investigate EBV-encoded BARF1-induced changes such as cell proliferation and cellular miRNA and protein expression.

## RESULTS

### Secreted BARF1 was detected in BARF1-expressing SNU 601 cells

**Figure 1:**
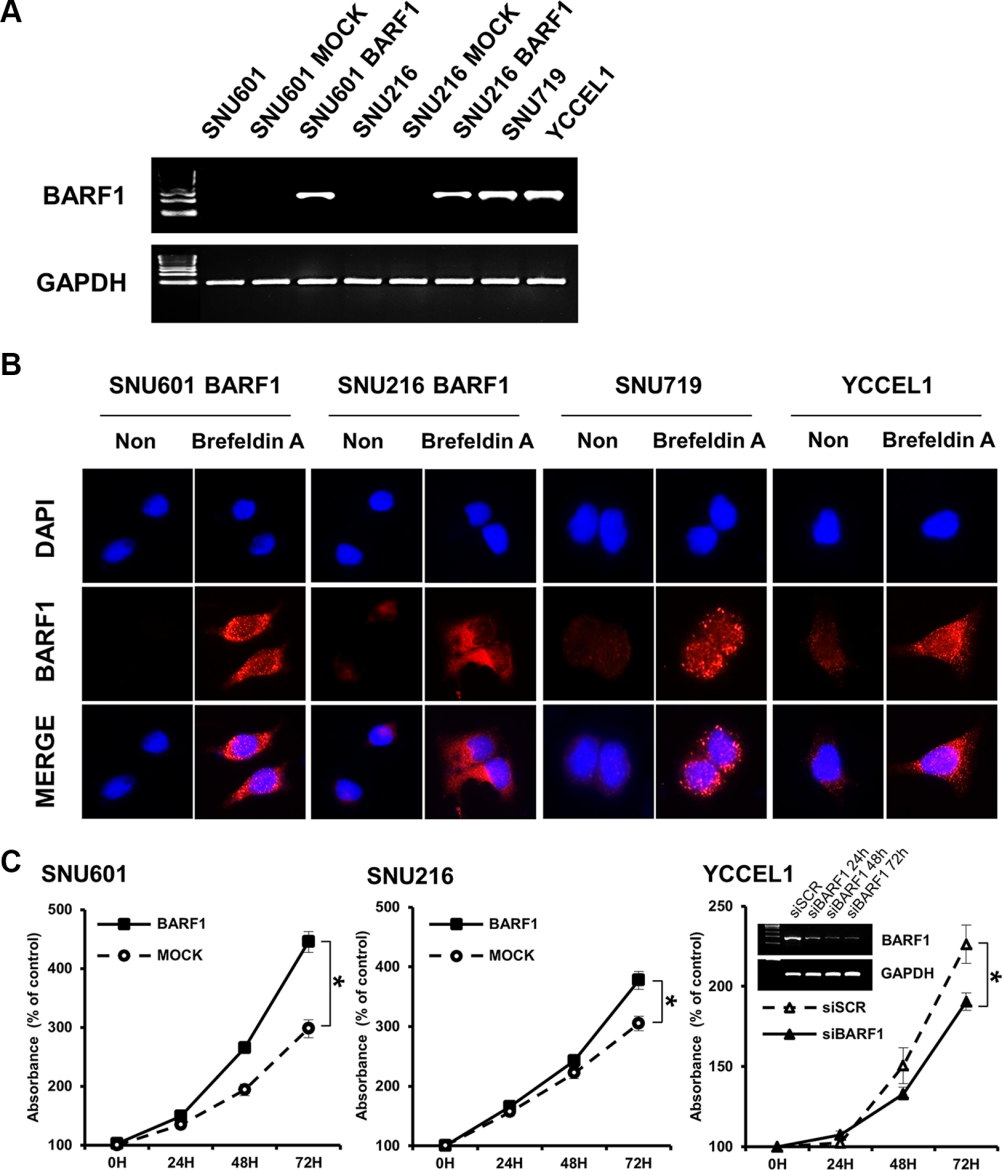
EBV BARF1 protein was mainly secreted and promoted cell proliferation (**A**) BARF1 mRNA was detected in SNU601 BARF1 and SNU216 BARF1 cells (stable transfection with BARF1) and in naturally Epstein-Barr virus (EBV)-infected stomach cancer cells (SNU719 and YCCEL1), whereas it was undetectable in SNU601 mock cells and SNU216 mock cells. (**B**) As seen by fluorescence microscopy, BARF1 protein was hardly observed in SNU601 BARF1 cells and was weakly detected in SNU216 BARF1, SNU719 and YCCEL1 cells, whereas BARF1 protein accumulated in cells that were treated with Brefeldin A. BARF1 antibody (MAb 6F4) was labeled in red, and nuclei were counterstained with DAPI (blue). (**C**) Cell proliferation was higher in SNU601 BARF1 and SNU216 BARF1 cells than in SNU601 mock cells and SNU216 mock cells, respectively, and lower in YCCEL1 cells transfected with BARF1-specific siRNA (siBARF1) than in YCCEL1 cells transfected with scrambled siRNA (siSCR) (*P < 0.05). All experiments were performed in triplicate.

BARF1 transcripts were detected in BARF1-expressing cells (SNU601 BARF1 and SNU216 BARF1) and in the naturally EBV-infected stomach cancer cell lines SNU719 [7, 8] and YCCEL1 [48], but not in SNU601 and SNU216 mock cells (transfected with an empty vector) (Figure 1A). BARF1 protein was almost undetectable in untreated SNU601 BARF1 cells and weakly detected in untreated SNU216 BARF1, SNU719 and YCCEL1 cells; however, BARF1 accumulated in cells treated with Brefeldin A (to block protein secretion), suggesting that translated BARF1 is mainly secreted (Figure 1B).

### BARF1 promoted stomach cancer cell proliferation

Both SNU601 BARF1 cells and SNU 216 BARF1 cells showed higher rates of cell proliferation than their mock cells (*P* < 0.05; Figure 1C). Conversely, YCCEL1 cells transfected with siRNA against BARF1 (siBARF1) showed a lower rate of cell proliferation than scrambled siRNA (siSCR)-transfected YCCEL1 cells (Figure 1C).

### BARF1 upregulated miR-146a-5p in an NFκB-dependent manner

**Figure 2:**
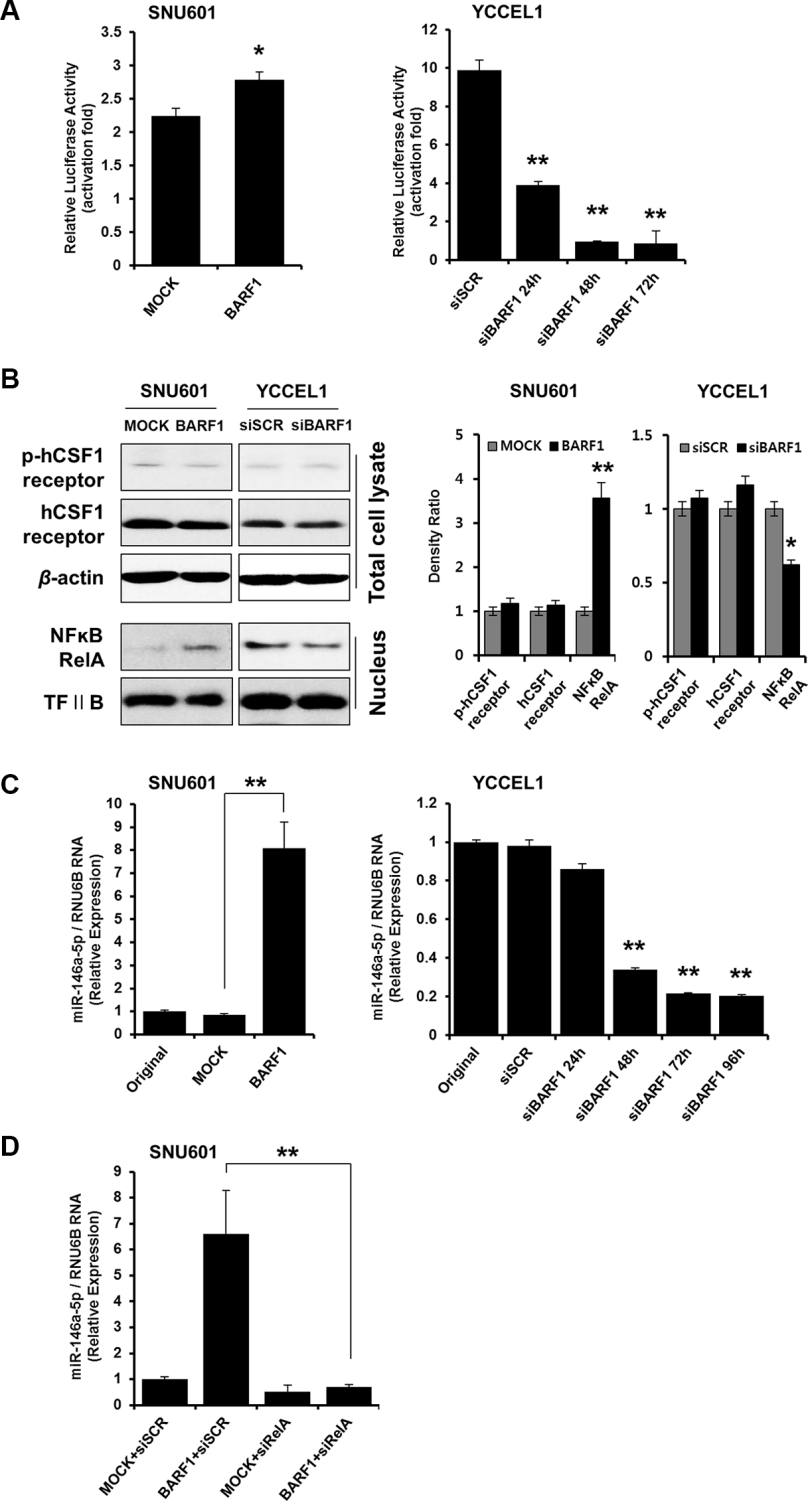
BARF1 upregulated miR-146a-5p in an NFκB-dependent manner (**A**) Cells were transfected with an NFκB-dependent luciferase reporter together with Renilla luciferase. After 72 h, NFκB activity was determined using a dual-luciferase assay. SNU610 BARF1 cells demonstrated higher NFκB transcriptional activity than SNU601 mock cells (*P < 0.05). YCCEL1 cells transfected with 20 nM BARF1-specific siRNA (siBARF1) showed lower NFκB transcriptional activity than YCCEL1 cells transfected with scrambled siRNA (siSCR) (**P < 0.01). (**B**) Phospho-hCSF1 receptor and hCSF1 receptor showed similar levels irrespective of BARF1 presence or knockdown, whereas NFκB RelA and miR-146a-5p increased in response to BARF1. (**C**) TaqMan quantitative real-time RT-PCR showed higher miR-146a-5p levels in SNU601 BARF1 cells than in SNU601 mock cells or untransfected SNU601 cells (**P < 0.01). Conversely, miR-146a-5p expression was markedly decreased in YCCEL1 cells transfected with BARF1-specific siRNA (siBARF1) compared with YCCEL1 cells transfected with scrambled siRNA (siSCR) or untransfected YCCEL1 cells (**P < 0.01). (**D**) SNU601 BARF1 cells were transfected with 20 nM NFκB RelA-specific siRNA (siRelA) or scrambled siRNA (siSCR). BARF1-induced miR-146a-5p upregulation was neutralized by NFκB RelA inhibition (**P < 0.01). All experiments were performed in triplicate.

To examine the mechanism underlying the cell proliferation effect of BARF1, we analyzed the potential role of NFκB. NFκB luciferase activity was higher in SNU601 BARF1 cells than in SNU601 mock cells (*P* < 0.05), and NFκB activity was lower in siBARF1-transfected YCCEL1 cells than in scrambled siRNA-transfected control YCCEL1 cells (*P* < 0.01) (Figure 2A). The levels of phospho-hCSF1 receptor and hCSF1 receptor were unaltered irrespective of BARF1 presence or knockdown, while BARF1 induced NFκB and miR-146a-5p upregulation (Figure 2B). We then examined the association of miR-146a-5p, a cellular miRNA, with NFκB. miR-146a-5p levels were significantly higher in SNU601 BARF1 cells than in SNU601 mock cells (*P* < 0.01), and miR-146a-5p was downregulated in siBARF1-transfected YCCEL1 cells compared with scrambled siRNA-transfected control (*P* < 0.01) (Figure 2C). Transfection of SNU601 BARF1 cells with NFκB RelA-specific siRNA suppressed the BARF1-induced upregulation of miR-146a-5p (Figure 2D). These results indicate that BARF1 increased the levels of NFκB RelA and upregulated miR-146a-5p expression in an NFκB-dependent manner.

### BARF1 downregulated SMAD4 in a miR-146a-5p-dependent manner, and SMAD4 was a direct target of miR-146a-5p in stomach cancer cells

**Figure 3:**
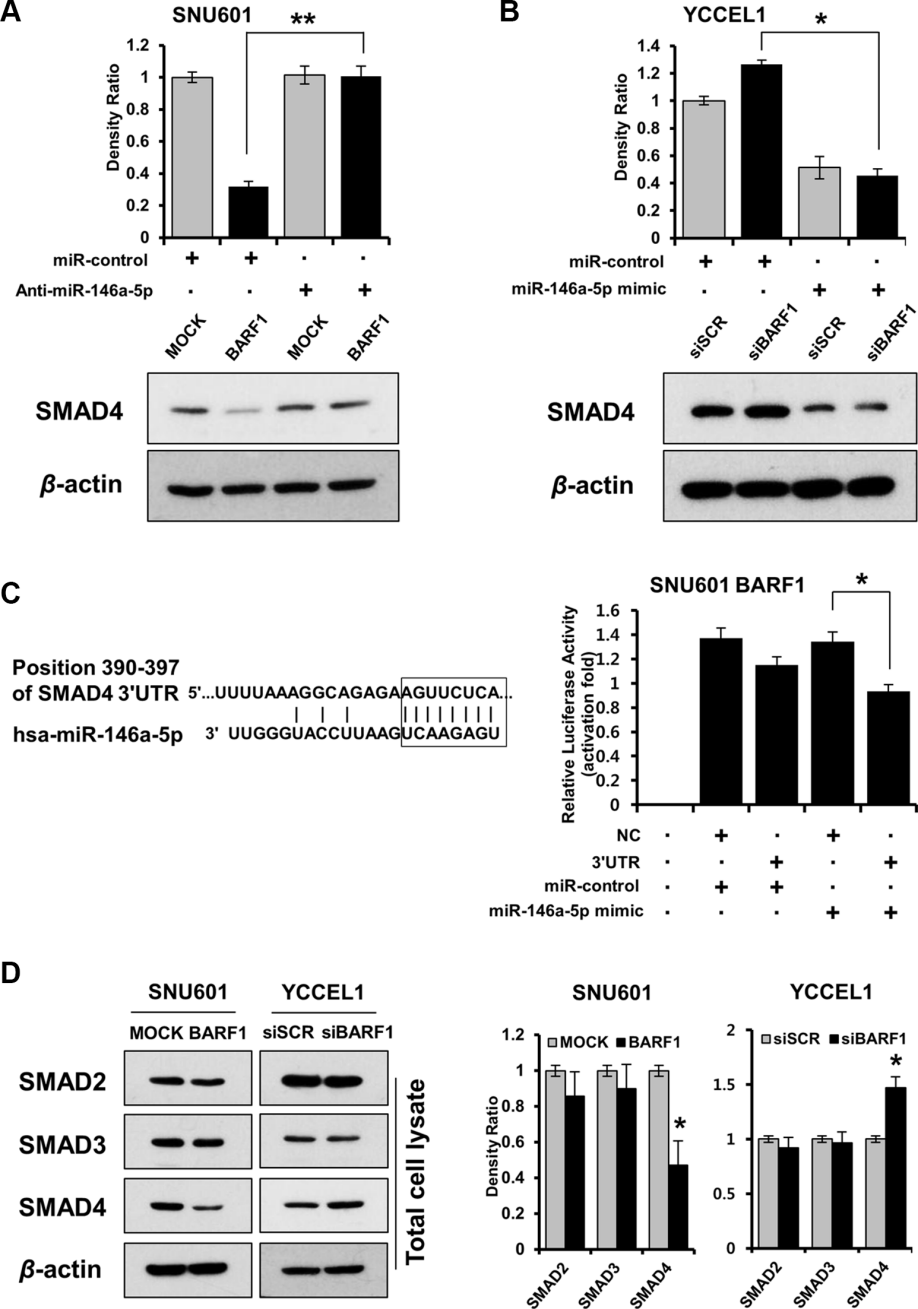
BARF1 downregulated SMAD4 in a miR-146a-5p-dependent manner, and SMAD4 was a direct target of miR-146a-5p (**A**) SMAD4 protein expression in SNU601 BARF1 cells was measured via western blotting after transfection with a miR-146a-5p inhibitor (anti-miR-146a) or a scrambled miRNA control (miR-control). SMAD4 protein level was downregulated in SNU601 BARF1 cells, and was restored by miR-146a-5p inhibition (*P < 0.05). (**B**) YCCEL1 cells (naturally EBV-infected stomach cancer) were transfected with 20 nM siRNAs (BARF1-specific or scrambled) and 50 nM miRNAs (miR-146a-5p mimic or miR-control). SMAD4 was upregulated in YCCEL1/siBARF1 cells and downregulated by the miR-146a-5p mimic (*P < 0.05). (**C**) The 8-mer sequence homology between miR-146a-5p and the 3′UTR of SMAD4 mRNA is shown. The mRNA transcript sequences were extracted from the website (http://www.ensembl.org). The luciferase assay revealed reduced reporter activity after co-transfection of SMAD4 3′UTR and miR-146a-5p in SNU601 BARF1 cells, compared with co-transfection of a negative control (NC, empty vector) and miR-146a-5p (*P < 0.05). All experiments were performed in triplicate. (**D**) BARF1 downregulated SMAD4 in total cell lysates, but had no effect on SMAD2 or SMAD3 expression. The bar graph shows the ratio of SMAD normalized to β-actin as determined by optical density measurement (*P < 0.05). All experiments were performed in triplicate.

To identify targets of miR-146a-5p, we used the prediction algorithm TargetScan Human 6.2 (http://www.targetscan.org), which showed that the 3′ UTRs of 200 mRNAs contained potential miR-146a-5p target sites. Among them, IL-1 receptor-associated kinase-1 (IRAK1) and SMAD4 were selected because of their role in NFκB activation [41, 42, 50]. Because BARF1 downregulated SMAD4 protein but had no effect on the level of IRAK1 (Supplementary Figure S2), we selected SMAD4 as a target of miR-146a-5p for subsequent analyses. miR-146a-5p knockdown by transfection with anti-miR-146a-5p restored SMAD4 protein levels in SNU601 BARF1 cells (Figure 3A). In YCCEL1 cells, siRNA-mediated silencing of BARF1 upregulated SMAD4 protein, whereas transfection with the miR-146a-5p mimic downregulated SMAD4 (Figure 3B). Furthermore, transient transfection of SNU601 BARF1 cells with the SMAD4 3′ UTR plasmid along with miR-146a-5p led to a significant decrease in relative luciferase activity, compared with the negative control (empty vector) along with miR-146a-5p (Figure 3C). The levels of SMAD2 and SMAD3 were not affected by BARF1 (Figure 3D).

### BARF1 downregulated nuclear SMAD4

**Figure 4:**
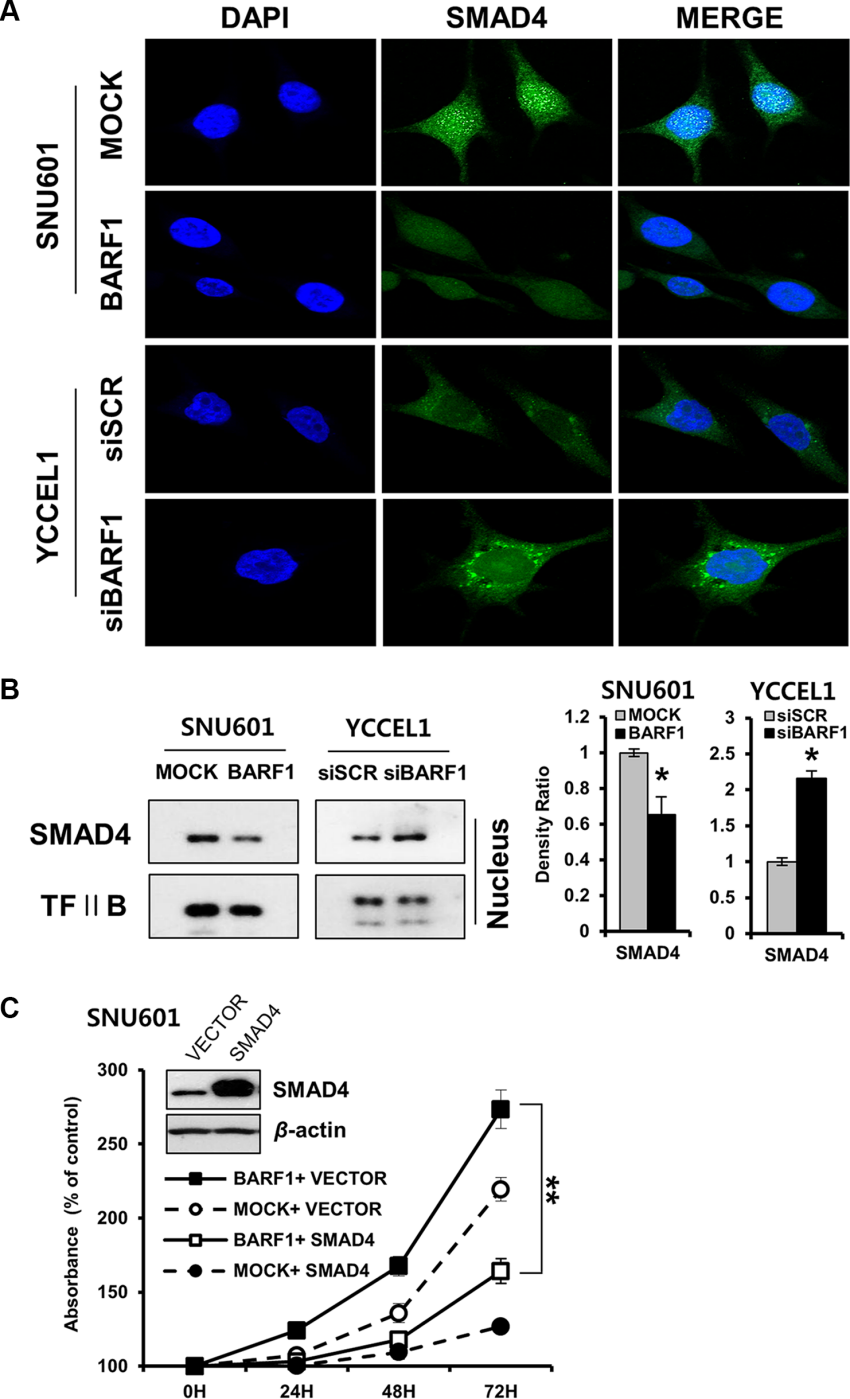
BARF1-mediated down regulation of nuclear SMAD4, and the effect of SMAD4 on cell proliferation (**A**) Immunofluorescence was visualized via confocal microscopy. BARF1 attenuated nuclear SMAD4 in SNU601 BARF1 cells, whereas BARF1 inhibition augmented nuclear SMAD4 in YCCEL1 cells transfected with siBARF1. Cells were labeled with SMAD4 antibody (green), and nuclei were counterstained with DAPI (blue). (**B**) Western blotting of nuclear extracts confirmed the BARF1-mediated downregulation of nuclear SMAD4 (*P < 0.05). TFII B was used as a loading control. (**C**) SNU601 BARF1 and SNU601 mock cells were transfected with pCMV-SMAD4 (SMAD4) or pCMV-empty vector (VECTOR). Cell proliferation was suppressed by pCMV-SMAD4 transfection compared with cells transfected with empty vector (**P < 0.01). All experiments were performed in triplicate.

Immunofluorescence analysis showed that nuclear SMAD4 protein levels were decreased in SNU601 BARF1 cells. Conversely, YCCEL1 cells transfected with siBARF1 showed increased nuclear SMAD4 protein levels (Figure 4A). This decrease in nuclear SMAD4 protein levels were confirmed via western blotting of nuclear protein extracts (*P* < 0.05) (Figure 4B).

### SMAD4 neutralized BARF1-induced cell proliferation

The rate of cell proliferation was lower in SNU601 BARF1 cells transfected with pCEP4-SMAD4 than in SNU601 BARF1 cells transfected with empty vector (*P* < 0.01), indicating that ectopic expression of SMAD4 counteracted the effect of BARF1 on promoting cell proliferation (Figure 4C). These results suggest that SMAD4 downregulation is critical for cell proliferation in BARF1-expressing cells.

### Verification of miR-146a-5p upregulation and NFκB immunohistochemical expression in EBV-positive stomach cancer tissues

**Figure 5:**
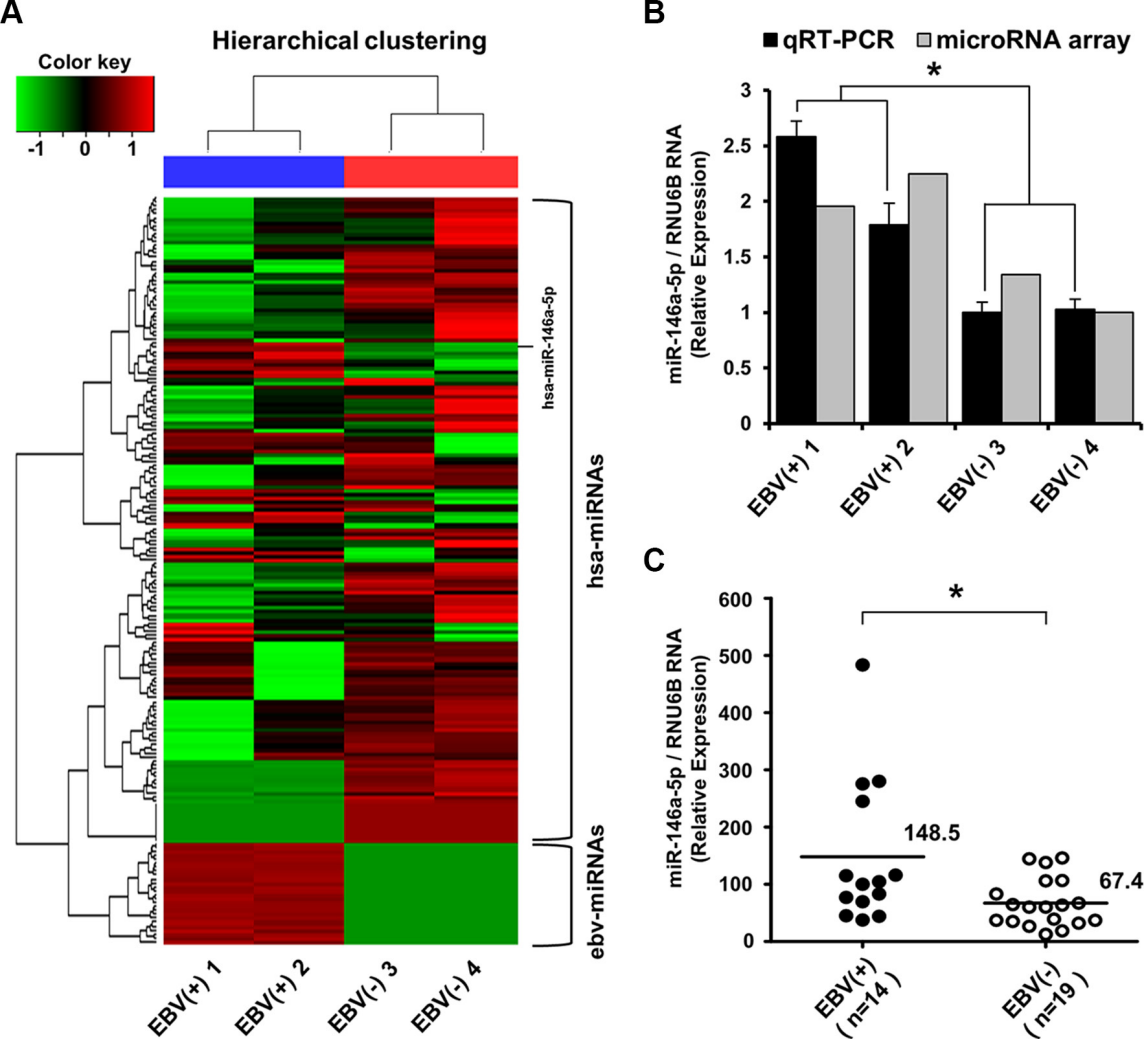
miRNA microarray expression profile and validation of miR-146a-5p level in surgically resected stomach cancer tissues (**A**) Heat map and hierarchical clustering of miRNA expression showed a clear contrast between EBV-positive and EBV-negative cancer groups, with 31 miRNAs being upregulated including miR-146a-5p. Tissue samples are denoted as EBV(+) 1 and EBV(+) 2 for EBV-infected stomach cancer tissues and EBV(−) 3 and EBV(−) 4 for EBV-negative stomach cancer cases. The upper horizontal section represents human cellular miRNAs (hsa-miRNAs), and the lower horizontal section indicates EBV miRNAs (ebv-miRNAs). The red color indicates the expression of miRNAs in the sample, and green indicates absent or downregulated miRNAs. (**B**) TaqMan quantitative real-time RT-PCR was used to validate miRNA microarray data. miR-146a-5p levels were higher in EBV-positive stomach cancer tissues than in EBV-negative stomach cancer tissues (*P < 0.05). (**C**) TaqMan quantitative real-time RT-PCR for miR-146a-5p was performed for an additional 14 cases of EBV-positive stomach cancer tissues and the other additional 19 EBV-negative stomach cancer tissues. The miR-146a-5p level in the EBV-positive stomach cancer group was higher than that in the EBV-negative stomach cancer group (*P < 0.05). RNU6B was used to normalize miR-146a-5p expression.

According to the miRNA microarray analysis, 139 cellular miRNAs were differentially expressed with a 1.5-fold difference between EBV-positive and EBV-negative stomach cancer tissues (Figure 5A). Thirty-one cellular miRNAs including miR-146a-5p were upregulated and 108 were downregulated in EBV-infected versus EBV-negative stomach cancer tissues (Supplementary Figure S3). The upregulation of miR-146a-5p in EBV-positive stomach cancer tissues was validated using TaqMan quantitative real-time RT-PCR (Figure 5B).

Additionally, in stomach cancer tissue surgically resected in 2012, miR-146a-5p was highly expressed in the EBV-positive stomach cancer group (*n* = 14) compared with the EBV-negative stomach cancer group (*n* = 19) (Figure 5C). Immunohistochemically, NFκB expression was more frequently observed with a marginal significance (*P* = 0.080) in the EBV-positive group, but not SMAD4 loss (Table 1).

**Table 1:** Comparison of NF*κ*B RelA, miR-146a-5p and SMAD4 between Epstein-Barr virus-positive and Epstein-Barr virus-negative stomach cancer

	EBV-positive	EBV-negative	
	(*n* = 14)	(*n* = 19)	*P* value
NF*κ*B RelA			0.080
negative	9 (67%)	17 (90%)	
positive	5 (33%)	2 (10%)	
miR-146a-5p			0.033
mean / median	148.5/102.3	67.4/60.4	
(range)	(38.0 ~ 483.6)	(13.0 ~ 146.4)	
SMAD4			0.580
loss	7 (50%)	10 (53%)	
preserved	7 (50%)	9 (47%)	

### SMAD4 nuclear loss tended to be associated with poor prognosis in EBV-positive, but not EBV-negative stomach cancer patients

**Figure 6:**
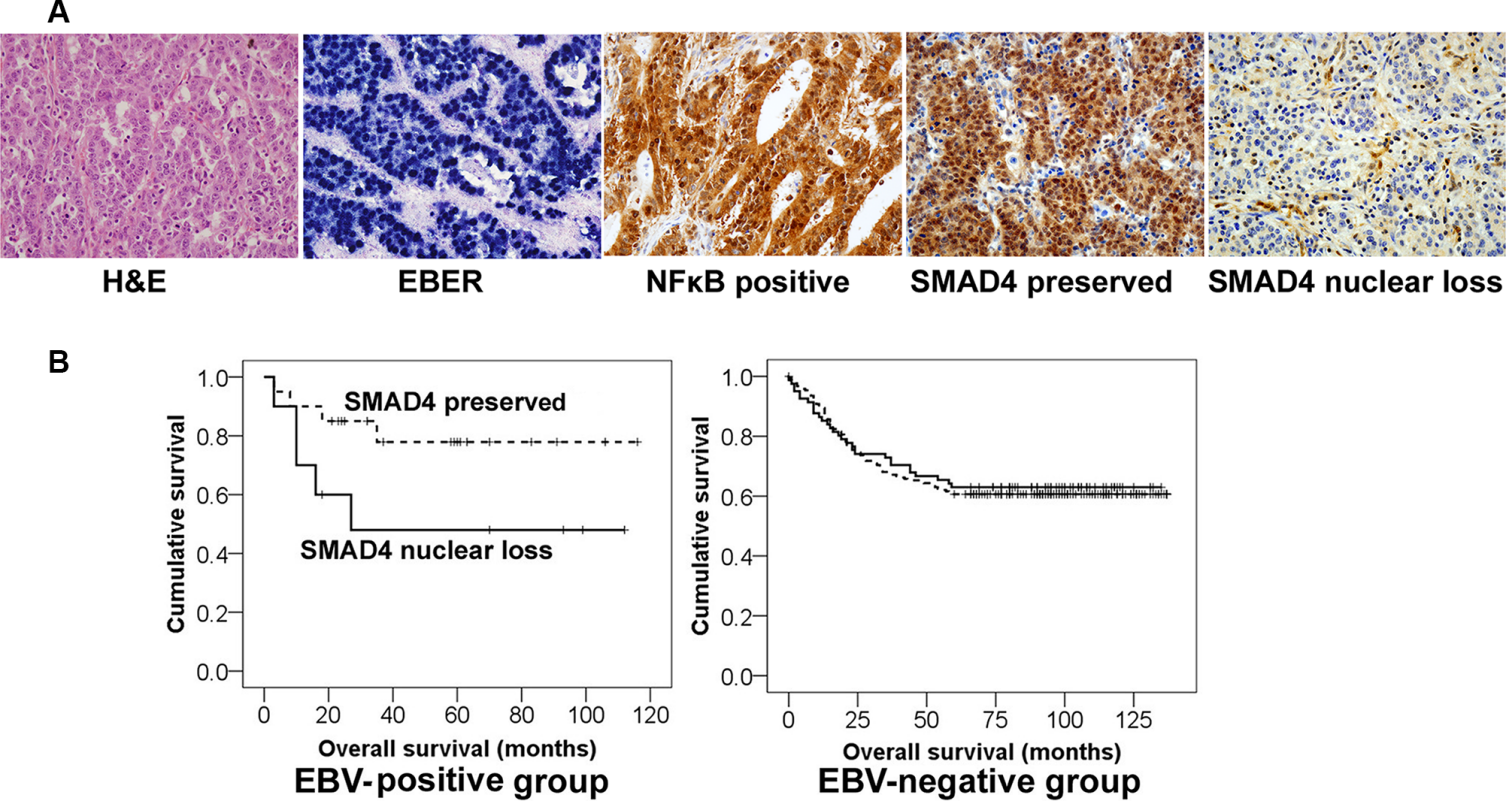
Different clinical implication of SMAD4 expression in EBV-positive and EBV-negative stomach cancer patients (**A**) Representative microscopic images of the histologic features of EBV-positive stomach cancer tissues. *In-situ* hybridization for EBV-encoded small RNAs (EBER) showed black signals in almost all of the cancer cell nuclei. NFκB and SMAD4 immunohistochemistry was evaluated only nuclear staining of cancer cells. (**B**) Kaplan-Meier survival curves. The ‘SMAD4 loss’ group (solid line) tended to show a worse survival rate than the ‘SMAD4 preserved’ group (dotted line) among EBV-positive cancer patients (*P* = 0.08), whereas SMAD4 had no prognostic significance in EBV-negative patients.

For the patient survival analysis, we chose stomach cancers that were surgically resected in 2000~2005. The mean follow-up period for patient outcome was 68 months (median: 77 months). Of 328 cases, thirty cases were EBV-positive and 298 were EBV-negative (Supplementary Table S1). Immunohistochemical analysis showed SMAD4 nuclear loss in 10 of 30 EBV-positive cancer patients, of whom five (50% of SMAD4 loss/EBV-positive) died. Meanwhile, SMAD4 nuclear loss was observed in 81 cases in the EBV-negative group, of whom 30 patients (37% of SMAD4 loss/EBV-negative) were dead. Statistically, the SMAD4 nuclear loss group tended to be associated with a worse survival rate compared with the SMAD4 preservation group among EBV-positive stomach cancer patients, but not statistically significant (*P* = 0.08), and SMAD4 showed no prognostic implication among EBV-negative stomach cancer patients (Table 2 and Figure 6).

**Table 2:** Differential effect of SMAD4 on patient survival in Epstein-Barr virus-positive and Epstein-Barr virus-negative stomach cancer

	SMAD4 nuclear loss	SMAD4 preserved	
EBV-positive (*n* = 30)	10	20	*P* value
Dead	5 (50%)	4 (20%)	0.08
Alive	5 (50%)	16 (80%)	
EBV-negative (*n*= 298)	81	217	*P* value
Dead	30 (37%)	85 (39%)	0.76
Alive	51 (63%)	132 (61%)	

## DISCUSSION

The results of the present study indicate that EBV-encoded BARF1 promotes cell proliferation in stomach cancer through a mechanism involving NF*κ*B and miR-146a-5p upregulation and SMAD4 downregulation. Previous work from our group showed that NFκB RelA protein expression is higher in EBV-positive than in EBV-negative stomach cancer [5, 7, 8], and that BARF1 promotes stomach cancer cell proliferation by upregulating NF*κ*B [5]. Since then, we have been further searching for the molecular link between BARF1 and cell proliferation. The present study showed that BARF1 upregulated NF*κ*B and induced NF*κ*B-dependent miR-146a-5p upregulation. Currently, no studies have investigated EBV-encoded BARF1-induced cellular miRNA alterations; Motsch et al. showed that EBV-encoded LMP1 upregulates miR-146a in an NFκB-dependent manner [35], which is consistent with the results of the present study regarding BARF1-induced NFκB/miR-146a upregulation. Unfortunately, the BARF1-induced NFκB /miR-146a /SMAD4 axis observed in cell line was not clearly recapitulated in tissues. BARF1 protein is expressed within EBV-positive tumor cells, after that, almost completely secreted out of tumor cells [18, 21, 22], and this was attested in the present study using a secretion blocker in cell lines. Accordingly, it is challenging to directly detect BARF1 protein in tissue sample. Instead, zur Hausen et al. demonstrated the existence of BARF1 [18, 21, 22]. in EBV-positive stomach cancer tissue using a BARF1 nucleic acid sequence-based amplification (NASBA) method [19]; thus, EBV-positive stomach cancer is thought to belong to a specialized EBV latency type I expressing BARF1 [3]. In addition, there may be limits to pursuing BARF1-specific effects on NF*κ*B, miR-146a and SMAD4 in tissues, as cancer-related cellular proteins are likely controlled by intricate pathways in cancer tissues. As such, BARF1 may have a restricted effect on NF*κ*B, miR-146a and SMAD4 in tissue.

The effect of BARF1 on NFκB and miR-146a in SNU601 BARF1 and naturally EBV-infected YCCEL1 cells may occur via intracellular signaling, rather than via the secretory pathway. The activation effect seems to be mediated by the N-terminal 1-20 AA domain of BARF1, which remains intracellular [18]. This idea is supported by the fact that NFκB and miR-146a levels decreased dramatically following BARF1-specific siRNA transfection in the present study. The hCSF1 receptor might not be involoved in the process of BARF1-induced NFκB and miR-146a-5p upregulation, because phospho-hCSF1 receptor and hCSF1 receptor levels were unchanged during NFκB and miR-146a-5p upregulation (Figure 2), and hCSF1 receptor blocking did not influence BARF1-induced NFκB upregulation in the present study (Supplementary Figure S4).

The present study suggests that BARF1 suppressed SMAD4 through NFκB-dependent miR-146a upregulation in stomach cancer cells. Previous studies found that the established viral oncogene LMP1 inhibits the transcriptional activity of SMAD via NFκB activation [46]. Moreover, the present study identified SMAD4 as a direct target of miR-146a in SNU601 BARF1 cells, providing the first experimental evidence in stomach cancer cells. Xiao et al. previously detected miR-146a upregulation in stomach cancer cell lines; however, the SMAD4 3′ UTR luciferase target assay results were obtained in HEK293 (human embryonic kidney) cells and not in stomach cancer cells [51]. Additionally, SMAD4 is a target of miR-146a in various malignancies, such as leukemia [39] and hepatocellular carcinoma [52].

The results of the present study suggest that SMAD4 may have different implication between EBV-positive stomach cancer and EBV-negative stomach cancer. SMAD4 nuclear loss, as detected by immunohistochemical staining in stomach cancer tissues, tended to be associated with poor survival rates in EBV-positive stomach cancer patients. Although the role of SMAD4 in the prognosis of EBV-positive stomach cancer patients has not been described previously, several reports have shown that SMAD4 loss is associated with poor prognosis in advanced stomach cancer patients [53–55]. In addition, SMAD4 functions as a tumor suppressor gene in stomach cancer [53, 56]. The altered SMAD4 nuclear accumulation that was detected in the present study supports the idea that SMAD4 is active and affects transcriptional activity when localized to the nucleus [43, 45]. With regard to a mechanism of SMAD4 nuclear loss, various mechanisms including dysregulation of nucleocytoplasmic shuttling, loss of heterozygosity, promoter hypermethylation and proteasome degradation are found in gastric carcinomas [53]. In particular, van Rees et al. reported that allelic loss of chromosome 18q (involving SMAD4 locus) was significantly less frequent in EBV-positive gastric carcinomas than in EBV-negative gastric carcinomas, which might be compensated for by a higher frequency of gene inactivation through promotor hypermethylation in EBV-positive gastric carcinomas [57].

In conclusion, EBV-encoded BARF1 promotes stomach cancer cell proliferation through a mechanism involving the upregulation of NF*κ*B and miR-146a and the downregulation of SMAD4. These results may help explain the molecular mechanisms of EBV-infected stomach cancer progression. Moreover, SMAD4 loss may be useful as a negative prognostic factor in EBV-positive stomach cancer patients.

## MATERIALS AND METHODS

### Cell culture and reagents

SNU719 (stomach cancer cell line naturally infected with EBV), SNU601 and SNU216 (EBV-negative stomach cancer cell lines) were purchased from the Korean Cell Line Bank (Seoul, Korea). Another EBV-infected stomach cancer cell line, YCCEL1, was supplied by Dr. Rha [48]. Stable BARF1-expressing stomach cancer cells, SNU601 BARF1 [5] and SNU216 BARF1 cells were established. In brief, BARF1 was cloned from the naturally EBV-infected stomach cancer cell line SNU719. A pCMV-Tag 2B/flag/BARF1 plasmid was introduced into EBV-negative stomach cancer cells (SNU601 and SNU216) and transfectants were selected with G418 (Invitrogen, Carlsbad, CA, USA) (Supplementary Figure S1). Cells were maintained in RPMI 1640 medium (Gibco BRL, Rockville, MD, USA) supplemented with 10% fetal bovine serum and antibiotics (100 U/mL penicillin and streptomycin) at 37°C in a 5% CO_2_ atmosphere. Brefeldin A was used to inhibit protein transport from the endoplasmic reticulum to the Golgi apparatus (Sigma-Aldrich, St. Louis, MO, USA).

### RNA isolation and reverse transcription-polymerase chain reaction (RT-PCR)

Total cellular RNA was prepared using an RNeasy Mini Kit (Qiagen, Hilden, Germany). Extracted RNA was treated for 20 min at 37°C with 10 units of DNase I (Roche, Basel, Switzerland) in the presence of RNase inhibitor (Roche) to remove residual genomic DNA. After inactivation at 75°C for 10 min, RNA samples were purified with an RNeasy Mini Kit (Qiagen) according to the manufacturer's recommendations. cDNA was synthesized from 1 μg of total RNA (extracted from each sample) using a high fidelity RT-PCR system. The forward and reverse primers for cDNA amplification were as follows: BARF1 forward, 5′-CGGGATCCATGGCCAGGTTCATC-3′, and reverse, 5′-CCGCTCGAGTCATTGCGACAAGTAT-3′; GAPDH forward, 5′-GAGTCAACGGATTTGGTCGT-3′, and reverse, 5′-TTGATTTTGGAGGGATCTCG-3′. The PCR conditions were as follows: 30 cycles each of denaturation at 94°C for 30 sec, annealing at 54°C for 30 sec, and extension 72°C for 1 min. PCR products were analysed on 2% agarose gels.

### Immunofluorescence assay

Cells seeded on coverslips were fixed for 10 min in 4% paraformaldehyde in 10 mmol/L piperazine- N,N′-bis (2-ethanesulfonic acid) (PIPES), pH 6.8, 10 mmol/L NaCl, 300 mmol/L sucrose, 3 mmol/L MgCl_2_, and 2 mmol/L EDTA. Cells were permeabilized for 10 min in Tris-buffered saline (TBS) with 0.75% Triton X-100 and blocked for 10 min in 5% bovine serum albumin and 0.1% Triton X-100 in TBS. Cells were then incubated with antibodies against BARF1 (MAb 6F4, 1:100) [5] and SMAD4 (sc-7966, 1:200 Santa Cruz Biotechnology, Santa Cruz, CA, USA) and Alexa Fluor 488 goat anti-mouse IgG (H+L) secondary antibody (Invitrogen). Nuclei were counterstained with 4′,6-diamidino-2-phenylindole (DAPI) (1 μg/mL). Stained cells were visualized on a Zeiss Observer.Z1 fluorescence microscope or a Zeiss LSM710 confocal system (Carl Zeiss Meditec, Jena, Germany).

### Cell proliferation assay

Cells were seeded into 96-well plates, treated as indicated, and incubated with the cell proliferation reagent Cell Counting Kit-8 (CCK-8; Dojindo Laboratories, Kumamoto, Japan) (10 μL of CCK-8) for 2 h. Absorbance was measured at 450 nm on a spectrophotometer (Spectramax 190; Molecular Devices, Sunnyvale, CA, USA).

### Quantitative real-time RT-PCR

Total cellular RNA including miRNA was isolated using the miRNeasy Mini Kit (Qiagen). miRNA cDNA synthesis was carried out with 10 ng of total RNA from each sample using the TaqMan MicroRNA Reverse Transcription Kit (Ambion Life Technologies, Grand Island, NY, USA). TaqMan quantitative real-time RT-PCR analysis was performed with an ABI 7900 Real-Time PCR System using the TaqMan miRNA assay (Applied Biosystems, Foster City, CA, USA) with the hsa-miR-146a-5p (MIMAT0000449) primer set according to the manufacturer's protocol. RNU6b (Applied Biosystems) was used as a loading control in the TaqMan microRNA assay.

### siRNA, miRNA and plasmid transfection

NFκB RelA-specific small interfering RNA (siRNA) and BARF1-specific siRNA (5′-GAGCCUCGGU CCAGAGAUUUU-3′) were synthesized by Dharmacon RNA Technologies (Dharmacon, Lafayette, CO, USA). A scrambled siRNA (Dharmacon) containing a random sequence of nucleotides without known specificity was used as a negative control.

The miR-146a-5p mimic (hsa-miR-146a-5p; MC10722), miR-146a-5p inhibitor (hsa-miR-146a-5p; MH10722), and scrambled miRNA control (miR-control) were purchased from Ambion Applied Biosystems (Ambion Life Technologies). Transfections were performed using Lipofectamine 2000 (Invitrogen). Cells were transfected with miR-146a-5p mimic, miR-146a-5p inhibitor, or miR-control at a final concentration of 50 nM. The pCEP4-SMAD4 plasmid was obtained from the nonprofit plasmid repository Addgene (Cambridge, MA, USA) and was donated by Dr. Vogelstein [49].

### Western blot and densitometric analyses

Protein concentrations were determined using a BCA protein assay kit (Merck, Gibbstown, NJ, USA). Proteins were separated by SDS-PAGE gels topped by a 5% stacking gel and then transferred onto reinforced PVDF membranes (Millipore, Bedford, MA, USA). After incubation to block non-specific sites, blots were incubated overnight with primary antibodies against NFκB RelA (sc-109, 1:500, Santa Cruz Biotechnology), phospho-hCSF1 receptor (1:1000, Cell Signaling, Beverly, MA, USA), hCSF1 receptor (1:1000, Cell Signaling), SMAD2 (D43B4, 1:1000, Cell Signaling), SMAD3 (C67H9, 1:1000, Cell Signaling), and SMAD4 (B-8, sc-7966, 1:1000, Santa Cruz Biotechnology) at 4°C. Blots were then washed and incubated for 30 min at room temperature with a horseradish peroxidase-conjugated anti-mouse secondary antibody (Abcam, Cambridge, UK). The antigen-antibody complexes were visualized using ECL-staining (Amersham, Arlington Heights, IL, USA) and expose to X-ray film. Antibodies against transcription factor IIB (sc-23875, 1:2000, Santa Cruz Biotechnology) and β-actin (AC -15, 1:10000, Abcam) were used to verify equal loading and transfer of nuclear and total proteins, respectively. Developed films were imaged using a GS-700 Imaging Densitometer (Bio-Rad, Hercules, CA, USA) and processed with Corel Photo Paint 7.0 to adjust image brightness and contrast. Densitometric evaluation was performed using Molecular Analyst Software (Bio-Rad), and normalization was performed using the corresponding controls depending on the specific analysis.

### Preparation of nuclear extracts

Cells were resuspended in 100 μL of lysis buffer A (10 mmol/L Tris, pH 8.0, 60 mmol/L NaCl, 1 mmol/L EDTA, 1 mmol/L dithiothreitol, 0.1% Nonidet P-40 and 1 mmol/L phenylmethylsulfonyl fluoride) and incubated on ice for 5 min. Nuclear pellets were immediately washed in 1 mL of lysis buffer A without Nonidet P-40, centrifuged, and resuspended in 50 μL of buffer B (200 mmol/L HEPES, pH 7.9, 0.75 mmol/L spermidine, 0.15 mmol/L spermine, 0.2 mmol/L EDTA, 2 mmol/L EGTA, 2 mmol/L dithiothreitol, 20% glycerol, 1 mmol/L phenylmethylsulfonyl fluoride, and 0.4 M NaCl).

### Luciferase activity assay

To measure NFκB activity, cells were transfected with a cis-reporter plasmid containing the luciferase reporter gene linked to five tandem NFκB binding sites (0.8 μg, pNFκB Luc vector; Stratagene, La Jolla, CA, USA) using Lipofectamine 2000 (Invitrogen). A Renilla luciferase control reporter vector (0.04 μg, pRL-SV40:E2231; Promega, Madison, WI, USA) was co-transfected to normalize for transfection efficiency. After 72 h, luciferase activity was measured using a Dual-Luciferase Reporter Assay System kit (Promega) and was normalized to Renilla luciferase activity.

For the SMAD4 3′UTR assay, SNU601 BARF1 cells were seeded into 12-well plates one day before transfection, and then co-transfected with 0.8 μg of pEZX-MT06 vector, containing either the SMAD4 3′UTR Renilla/firefly dual-luciferase reporter plasmid (GeneCopoeia, Rockville, MD, USA) or empty vector (GeneCopoeia), along with 50 nM of miR-146a-5p mimic or scrambled miRNA control (miR-control). After 48 h, luciferase activity was measured using the dual-luciferase assay kit (Promega). Renilla luciferase activity was normalized to firefly luciferase activity. All assays were conducted in triplicate, and each transfection with reporter plasmid was carried out on a different day.

### miRNA microarray and data analysis

Formalin-fixed paraffin-embedded tissue samples were cut with a microtome. Cut sections were placed in 1.5 mL tubes, and total RNA including miRNA was extracted using the miRNeasy FFPE kit (Qiagen) according to the manufacturer's instructions. The tissue lysate was treated with DNase I (Roche) to exclude DNA contamination. The miRNA expression profiles of stomach cancer tissue were established using the SurePrint G3 Human miRNA Microarray, Release 16.0, 8x60K (Agilent Technologies, Santa Clara, CA, USA), which is based on miRBase v16.0 (http://www.mirbase.org) and contains a total of 1205 human and 144 human viral miRNAs. All procedures were performed according to the manufacturer's recommendations. To analyse the differentially expressed miRNAs, quantile normalization was performed to standardize the data across the samples. Two cases of EBV-positive stomach cancer were grouped and analyzed against another two cases of EBV-negative stomach cancer using a fold-change cut-off of 1.5. Hierarchical clustering was then performed using the Euclidean distance metric and the complete linkage rule.

### RNA *in-situ* hybridization and immunohistochemistry of surgically resected stomach cancer tissues

For patient prognosis study, we used formalin-fixed paraffin-embedded tissues from 328 stomach cancer patients who had undergone surgery at the Seoul National University Boramae Hospital. In total, 30 cases of EBV-positive stomach cancer were included in the study: 13 (4.2%) were from 311 consecutive surgeries that were performed between 2000 and 2005, and 17 were obtained from surgical cases in 2006~2010. For miRNA study, more recent formalin-fixed paraffin-embedded tissues should be needed. Accordingly, we selected stomach cancer tissues surgically resected in 2012, in which there were 14 EBV-positive and 19 EBV-negative cases. The retrospective study protocol was reviewed and approved by the Institutional Review Board of the Seoul National University Boramae Hospital under conditions of anonymity (IRB No. 20110318/06-2011-40/106 & 20150907/16-2015-124/101).

*In-situ* hybridization for EBV-encoded small RNAs (EBERs) was performed with the Discovery XT automated IHC/ISH stainer (Ventana Medical Systems, Tucson, AZ, USA), according to the manufacturer's instructions. A fluorescein-conjugated EBV oligonucleotide probe for EBERs was used (Novocastra, Newcastle-upon-Tyne, UK). Black or dark navy-colored signals at the hybridization site, detected by light microscopy, were interpreted as EBV-carrying cells. Rarely, reactive lymphocytes showed black signals, but only signals within tumor cell nuclei were considered positive. All of EBV-positive cases showed black signals in almost all cancer cell nuclei.

Immunohistochemistry for NF*κ*B and SMAD4 was performed with the Bond-Max automated immunostainer (Leica Microsystems, Nussloch, Germany). The tissue was incubated with anti-NFκB p65 (C-20) (sc-372, 1:150, Santa Cruz Biotechnology) or anti-SMAD4 antibody (B-8, sc-7966, 1:200, Santa Cruz Biotechnology), and the antigen was detected with the Bond Polymer Refine Detection Kit DC9800 (Leica Microsystems). Immunohistochemical staining was evaluated by two pathologists (M.S.C. & S-j.B.). Staining intensity was scored as 0 (no staining), 1+ (weak intensity), 2+ (moderate intensity), or 3+ (strong intensity). For NFκB, 2+ or 3+ staining in ≥ 5% of cancer cell nuclei was considered positive [7, 8]. Regarding SMAD4, cases showing 2+ or 3+ staining in more than 10% of the cancer cell nuclei were deemed as ‘SMAD4 preserved,’ and the remaining cases were defined as ‘SMAD4 loss’ [58].

### Statistical analysis

A chi-square test, Pearson's test, Kendall's Tau-b correlation analyses, Mann-Whitney U test and Kaplan–Meier survival analysis were performed with IBM^®^ SPSS^®^ Statistics 20.0 (IBM Inc., Chicago, IL, USA). A *P* value < 0.05 was considered statistically significant.

## SUPPLEMENTARY MATERIALS


