# Supplementary material for: Epstein-Barr virus BARF1-induced NFκB/miR-146a/SMAD4 alterations in stomach cancer cells
Source: Oncotarget. 2016 Jul 9;7(50):82213–27. doi: 10.18632/oncotarget.10511 (PMC5347686; doi:10.18632/oncotarget.10511)
Supplement: Supplementary file 1 [file oncotarget-07-82213-s001.pdf]

Epstein-Barr virus BARF1-induced NFκB/miR-146a/SMAD4 alterations in stomach cancer cells

Supplementary Materials

MARFIAQLLL LASCVAAGQA VTAFLGERVT LTSYWRRVSL GPEIEVSWFK
LGPGEEQVLI GRMHHDVIFI EWPFRGFFDI HRSANTFFLV VTAANISHDG
NYLCRMKLGE TEVTKQEHLS VVKPLTLSVH SERSQFPDFS VLTVTCTVNA
FPHPHVQWLM PEGVEPAPTA ANGGVMKEKD GSLSVAVDLS LPKPWHLPVT
CVGKNDKEEA HGVYVSGYLS Q

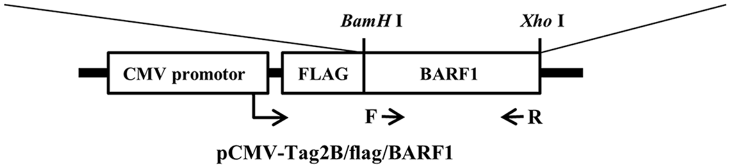

Supplementary Figure S1: BARF1 cloning. The BARF1 gene was cloned from a SNU719 (naturally EBV-infected stomach cancer cell line) cDNA library, which had the same peptide sequences as BARF1 from the B95.8 cell line. Peptide sequence data were based on resources from The National Center for Biotechnology Information (http://www.ncbi.nlm.nih.gov/protein/YP_401719.1).

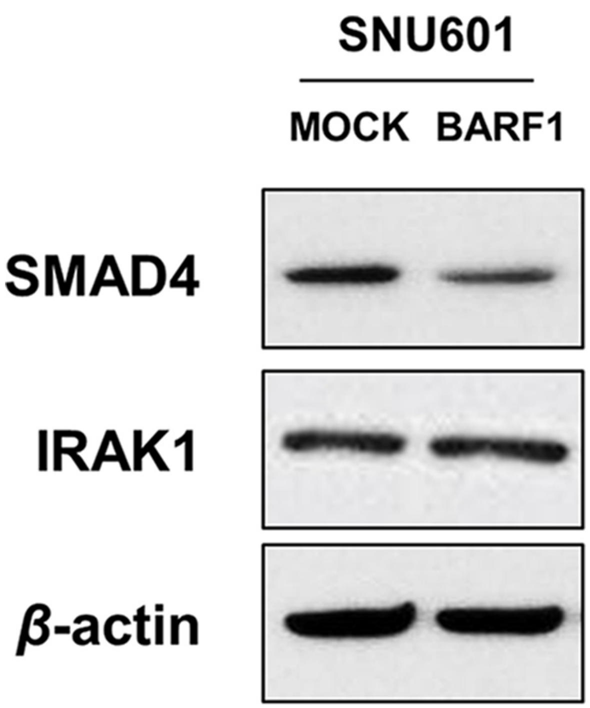

Supplementary Figure S2: SMAD4 and IRAK1 levels in BARF1-expressing cells. SMAD4 protein was downregulated by BARF1, whereas IRAK1 was not altered by BARF1.

A

MicroRNA Reporter	p-value	EBV(+) Mean	EBV(-) Mean	EBV(+)/EBV(-)
hsa-miR-223-3p	0.020	933.42	233.70	4.23
hsa-miR-34a-5p	0.081	497.60	236.33	2.43
hsa-miR-142-3p	0.087	1026.40	537.11	2.39
hsa-miR-1274a	0.231	832.42	372.03	2.28
hsa-miR-424-5p	0.077	64.46	31.57	2.23
hsa-miR-451a	0.148	1876.59	1111.41	2.16
hsa-miR-15b-5p	0.089	215.57	124.89	2.14
hsa-miR-1260a	0.230	2091.52	1002.97	2.13
hsa-miR-155-5p	0.091	190.64	90.23	2.12
hsa-miR-146b-5p	0.117	166.35	83.57	2.08
hsa-miR-133b	0.096	78.60	36.11	2.04
hsa-miR-3651	0.057	136.83	71.49	2.01
hsa-miR-15a-5p	0.113	215.71	131.76	1.93
hsa-miR-1280	0.131	1001.71	580.96	1.92
hsa-miR-16-5p	0.125	1230.26	795.46	1.91
hsa-miR-1	0.273	62.26	21.60	1.78
hsa-miR-142-5p	0.084	193.69	113.44	1.77
hsa-miR-1260b	0.279	1049.55	624.39	1.76
hsa-miR-27a-3p	0.043	847.76	550.69	1.74
hsa-miR-222-3p	0.025	131.17	68.62	1.71
hsa-let-7f-5p	0.193	1112.87	969.13	1.70
hsa-let-7g-5p	0.216	586.62	479.65	1.66
hsa-miR-146a-5p	0.034	150.43	98.61	1.64
hsa-miR-132-3p	0.056	27.67	12.76	1.62
hsa-miR-103a-3p	0.179	391.38	334.99	1.60
hsa-miR-4284	0.130	2388.45	1785.41	1.58
hsa-miR-185-5p	0.208	39.56	24.60	1.56
hsa-miR-20a-5p	0.264	266.19	216.37	1.55
hsa-miR-24-3p	0.039	756.96	597.99	1.52
hsa-miR-107	0.187	298.28	257.52	1.52
hsa-miR-221-3p	0.030	84.82	55.03	1.51

B

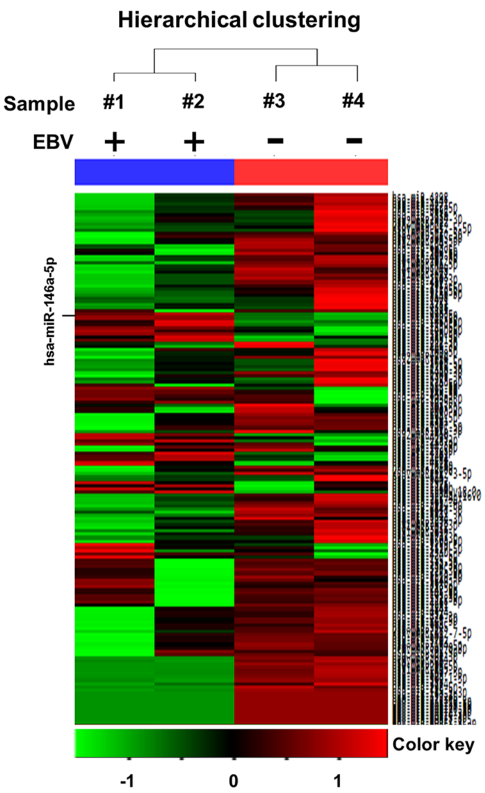

Supplementary Figure S3: The miRNA microarray data. (**A**) Thirty-one cellular miRNAs were upregulated in EBV-infected stomach cancer tissues compared with EBV-negative stomach cancer tissues. Mean signal intensities and log-transformed ratios are shown. (**B**) miR-146a-5p was marked in a heat map of unsupervised clustering composed of only the human miRNAs data.

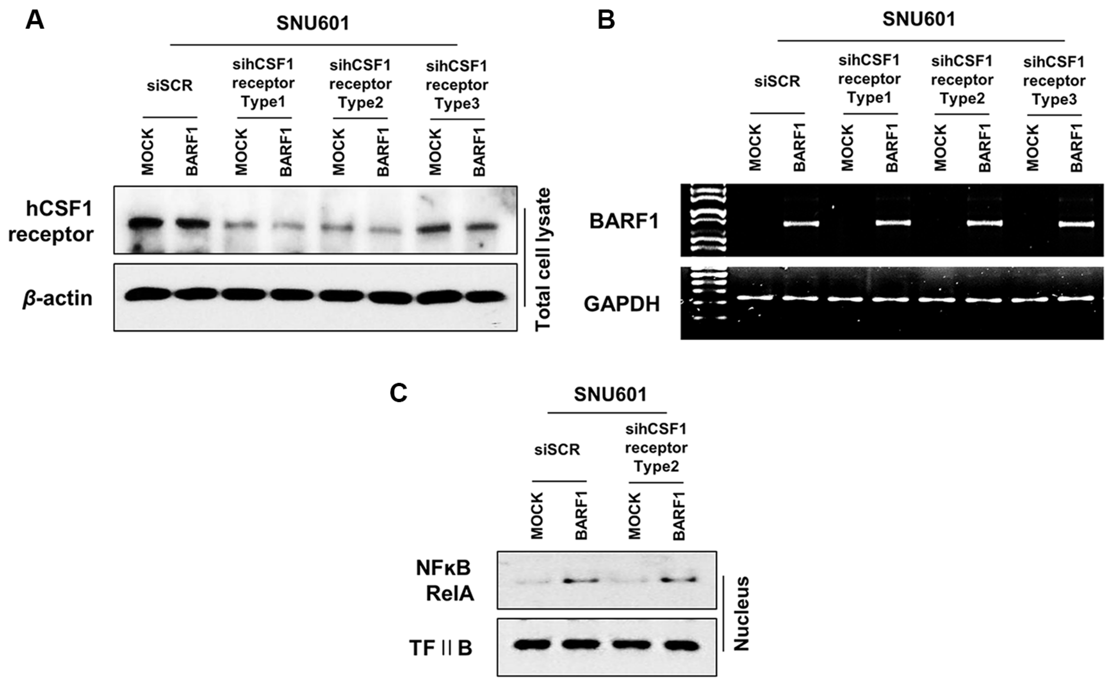

Supplementary Figure S4: Analysis of SNU601 transfected with hCSF1 receptor-specific siRNA. (**A**) The hCSF1 receptor expression was drastically reduced by transfection of hCSF1 receptor-specific siRNA-Type1 and -Type 2 in both of SNU601 mock and SNU601 BARF1 cells. (**B**) All three types of hCSF1 receptor-specific siRNA did not disturb BARF1, and it was reconfirmed by further inspection using MultAlin (http://multalin.toulouse.inra.fr/multalin/). (**C**) BARF1-induced NFκB upregulation was observed irrespective of hCSF1R blocking. All experiments were performed in triplicate. CSF1 receptor-specific small interfering RNA (siRNA)-Type 1, -Type 2 and -Type 3 were synthesized by Genolution Pharmaceuticals (Genolution, Seoul, Korea). A scrambled siRNA (Genolution) containing a random sequence of nucleotides with no known specificity was used as a negative control. Cells were transfected with siRNA at a final concentration of 25 nM. Each siRNA sequence was: Type 1 (sense, 5′-GCGUUGAUGUUAACUUUGAUU-3′; antisense 5′-UCAAAGUUAACAUCAACGCUU-3′), Type 2 (sense, 5′-CAACAAUCUGACUUUCAUAUU-3′; antisense 5′-UAUGAAAGUCAGAUUGUUGUU-3′), Type 3 (sense, 5′-CGAUCAAGUAGAUUUCCAAUU-3′; antisense 5′-UUGGAAAUCUACUUGAUCGUU-3′) and scrambled siRNA (sense, 5′-CCUCGUGCCGUUCCAUCAGGUAGUU-3′; antisense 5′-CUACCUGAUGGAACGGCACGAGGUU-3′).

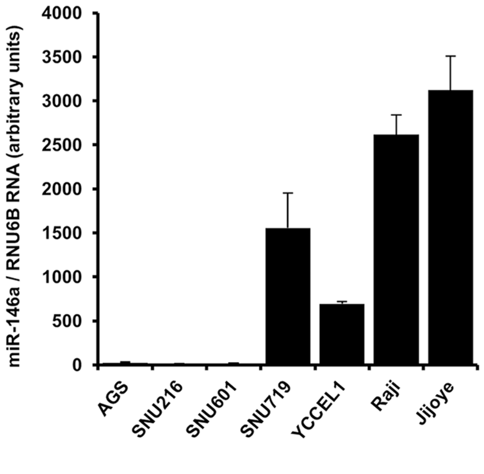

Supplementary Figure S5: Endogenous miR-146a-5p levels in various cell lines. miR-146a-5p levels were noticeably higher in EBV-infected cells than in EBV-negative cells. AGS, SNU216 and SNU601 are EBV-negative stomach cancer cells. SNU719 and YCCEL1 are EBV-infected stomach cancer cells. Raji and Jijoye are EBV-infected B cells.

Supplementary Table S1: Clinicopathological and immunohistochemical features of 328 cases of consecutive surgically resected-stomach cancer

	EBV-positive<br>(*n* = 30)	EBV-negative<br>(*n* = 298)	***P* value**
Age (yrs), mean (range)	62.6 (35-84)	62.8 (33-86)	not significant
Sex			0.023
male	26 (65%)	196 (66%)	
female	4 (35%)	102 (34%)	
Tumor location			< 0.001
low 1/3	7 (23%)	179 (60%)	
middle 1/3	11 (37%)	100(33%)	
upper 1/3	11 (37%)	8 (3%)	
whole	1 (3%)	11 (4%)	
Lauren classification			< 0.001
intestinal	4 (13%)	150 (50%)	
diffuse	23 (77%)	110 (37%)	
mixed	3 (10%)	38 (13%)	
Cancer stage*			0.002
Stage IA, IB	5 (17%)	130 (44%)	
Stage IIA, IIB	13 (43%)	53 (18%)	
Stage IIIA, IIIB, IIIC	10 (33%)	105(35%)	
Stage IV	2 (7%)	10 (3%)	
Patient survival			not significant
Alive	20 (67%)	164 (55%)	
Dead	10 (33%)	134 (45%)	
NFκB RelA			0.048
negative	20 (67%)	246 (83%)	
positive	10 (33%)	52 (17%)	
SMAD4			not significant
loss	10 (33%)	81 (27%)	
preserved	20 (67%)	217 (73%)	

*Cancer stage was determined via pathological assessment of TNM (tumor invasion depth-regional lymph node metastasis-distant metastasis) according to the American Joint Committee on Cancer 7th edition.
